# Small bowel obstruction due to a meat bolus bezoar: the second case report in literature

**DOI:** 10.1097/MS9.0000000000001633

**Published:** 2024-01-03

**Authors:** Ammar Albostani, Fadi Kfelati, Wafaa Alsaadi, Rufaida Ahmad Faraman, Aasem Farman

**Affiliations:** a University of Aleppo, Faculty of Medicine, Aleppo; b General Surgery Department, Al-Mouwassat University Hospital, Damascus University, Damascus, Syria

**Keywords:** Bezoar, case report, meat bolus, small bowel obstruction, surgery

## Abstract

**Introduction:**

Small bowel obstruction is a difficult emergency condition that may be caused due to many factors. However, bezoar-induced small bowel obstruction accounts for only 0.4–4.8% of all intestinal impaction patients. Bezoars are an entity of undigested materials classified into four types: phytobezoar, trichobezoar, pharmacobezoar, lactobezoar. Meat bolus bezoar is not named under any of these classifications.

**Case presentation:**

A 75-year-old man presented with abdominal distention, vomiting, and constipation. On radiological imaging, a mass in the terminal ileum was detected. After 2 days of ineffective conservative therapy, the authors decided to perform an open surgery. Enterotomy approach was chosen after failing to milk the object into the colons. The foreign body impacting the bowels was identified as a meat bolus bezoar. The patient improved after the surgery. The authors recorded no recurrence or complications with our patient after 18 months of follow-up.

**Discussion:**

Patients with small bowel obstruction usually present with acute abdominal pain and distension despite the blockage cause. Computed tomography is the most effective diagnostic tool in such cases. In bezoar-induced intestinal blockage, surgical management is mandatory if conservative therapy fails.

**Conclusion:**

It is important to consider bezoar-induced small bowel obstruction as a potential cause of impaction in cases of acute abdominal pain accompanied with risk factors of bezoar formation, despite the bezoar type.

## Introduction

Highlights

- Bezoar-induced small bowel obstruction is a rare condition reported in only a few cases. Bezoars are classified into four types, but meat bolus bezoars are not categorized under any of them.
- Computed tomography is considered the golden standard for identifying the characteristics of the obstructing objects within the intestines. Conservative therapy is usually ineffective in improving such conditions, making a surgical approach potentially mandatory.
- This paper presents the management of a case of a 75-year-old man with small bowel obstruction. The aim of this study is to discuss the types of bezoar-induced small bowel obstruction, associated symptoms, clinical and radiological findings, as well as therapeutic and surgical approaches in managing such cases.

Small bowel obstruction (SBO) is a medical emergency condition that can be challenging to diagnose and manage. Numerous factors and etiologies can lead to SBO, including postoperative adhesions as the most common cause, hernias, intussusception, and tumours. Bezoar-induced small bowel obstruction (BI-SBO) is a rare condition reported in only 0.4–4.8% of all SBO patients, being the fifth most frequent cause^1,2^.

The term “Bezoar” refers to a concreted entity of poorly or undigested foreign materials or food that are either accidentally or intentionally ingested, and accumulates within the gastrointestinal tract (GIT), occasionally causing GIT blockage^2–4^. In the literature, four types of bezoars are reported: phytobezoar (plant remnants), trichobezoar (hairball), pharmacobezoar (medications), and lactobezoar (mucus mass in milk-fed infants)^2,3,5,6^.

In this paper, we present the second case report in medical literature, to our knowledge, of terminal ileum obstruction due to a meat bolus bezoar. Moreover, it is worth noting that meat bolus bezoar is not classified under any of the established bezoar categories previously mentioned. We highlighted the challenges encountered in managing this unique case as per the SCARE 2020 Guidelines^7^.

## Case presentation

**Figure 1:**
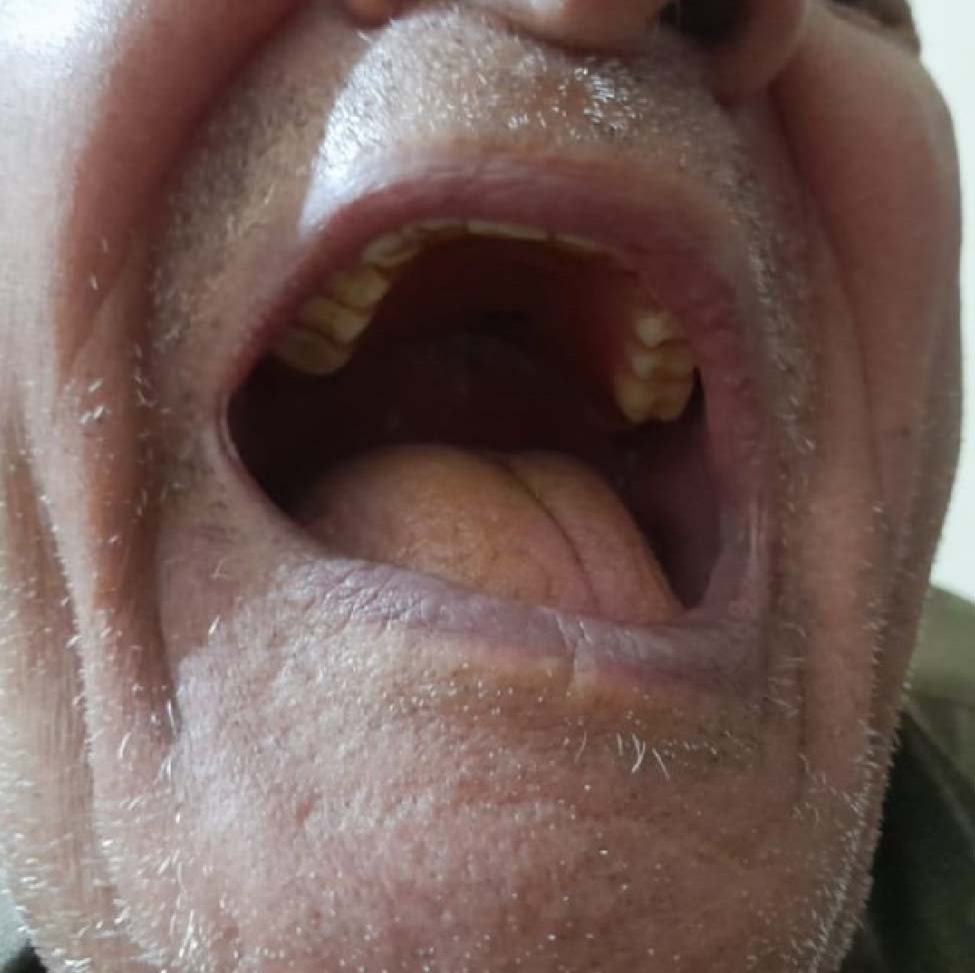
The patient’s mouth while wearing his dental prosthesis revealing his poor dental health that causes inadequate mastication.

A 75-year-old Syrian man presented to the emergency department with a chief complaint of abdominal distention, vomiting, and 3-day history of gas and stool constipation. His vital signs at the time of admission were as follows: Blood Pressure: 140/80 mmHg, Heart Rate: 83 pulse per min, Oxygenation: 93%. His general laboratory tests were within normal ranges. The patient had a medical history of diabetes mellitus type II, poor dental health (Fig. 1) as he wears a dental prosthesis, and had a surgical history of a right inguinal hernia repair ~40 years ago as he recalled. One day before admission, a physician prescribed laxatives (lactulose) to the patient, but his condition did not improve. On physical examination in our department, a severe abdominal distention was noticed.

**Figure 2:**
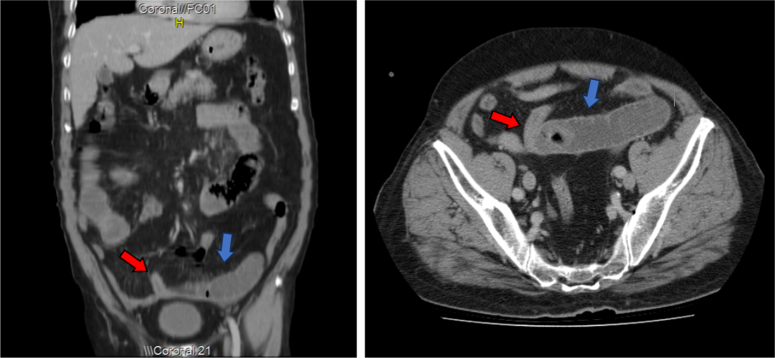
The object obstructing the bowels on the patient’s computed tomography scan of his pelvis and abdomen. (Red arrows) with the transitional dilated zone in intestinal loops (blue arrows).

We requested a simple abdominal X-ray (AXR) which revealed loops with air-fluid levels in the small intestine. For further investigation, an abdominal and pelvic computed tomography (CT) scan was obtained, which revealed a transitional dilated zone in the small intestines followed by a constricted area (Fig. 2). The patient was diagnosed with small bowel obstruction. However, radiological imaging did not identify the cause of impaction. Therefore, he was referred to conservative, nonsurgical, treatment with careful monitoring: nothing by mouth (NPO), intravenous fluids to correct the electrolyte imbalance, and antibiotics. After 2 days of conservative treatment, the patient’s state did not stabilize. Therefore, surgical intervention was mandatory.

**Figure 3:**
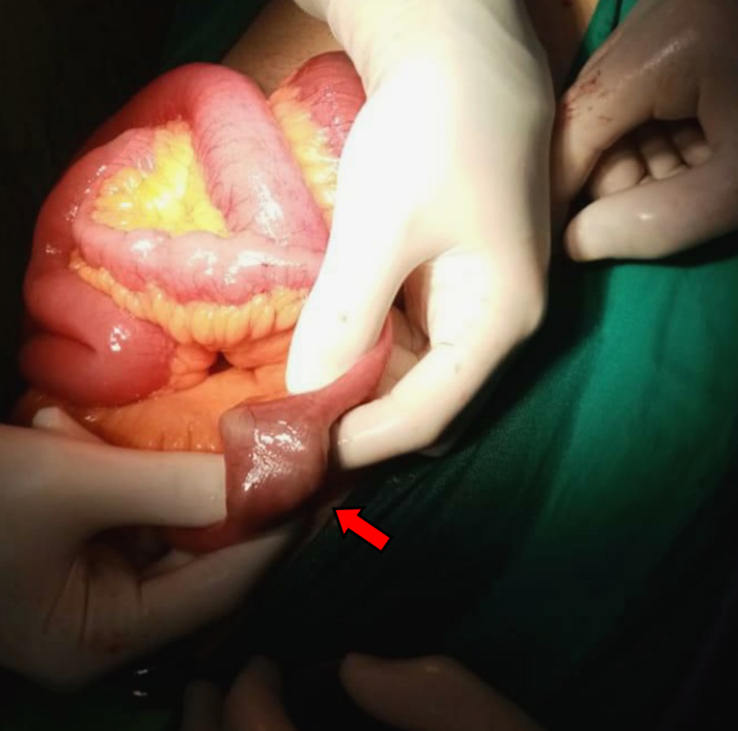
The obstruction area in the terminal ileum where the object is trapped in the lumen during surgery (red arrow).

**Figure 4:**
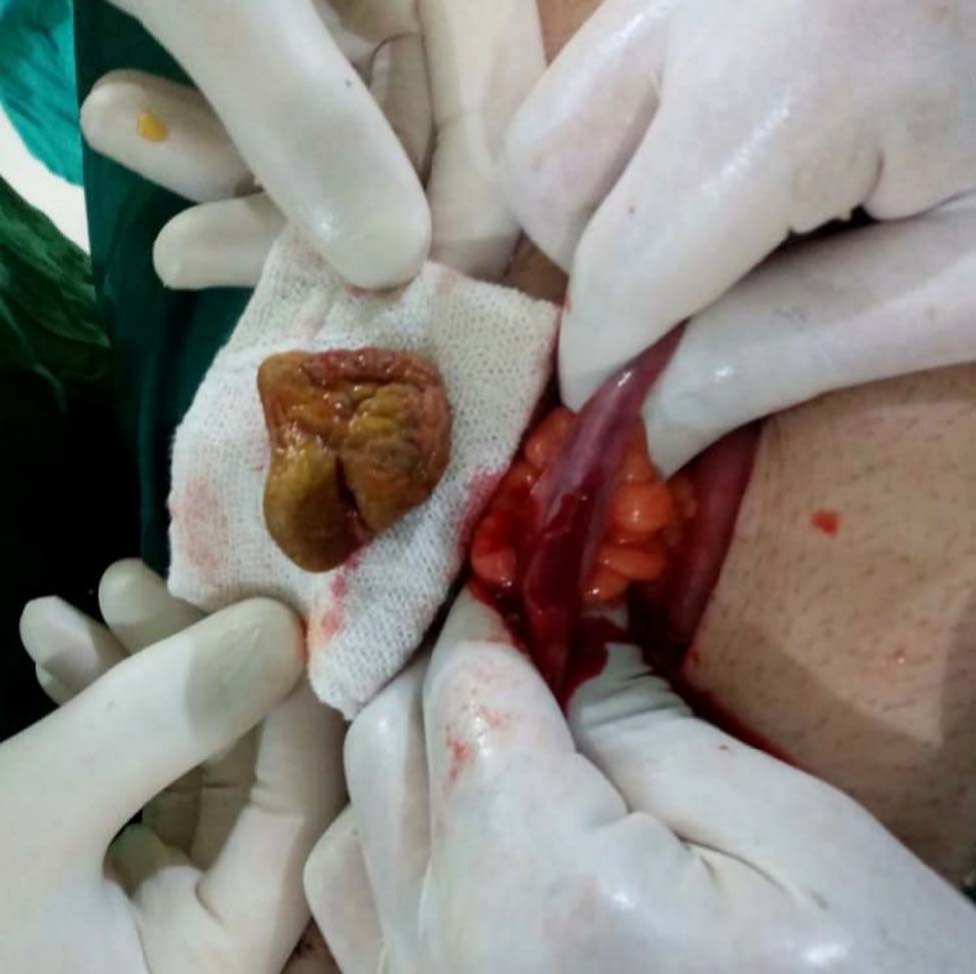
The meat bolus bezoar after surgical removal.

The surgeons opened the abdomen with a midline incision below the umbilicus. The examination of intestines revealed a foreign body obstructing the terminal ileum, located ~100 cm proximal to ileocecal junction (Fig. 3). Surgeons unsuccessfully tried to milk the bowels by pushing the foreign body into the colon. Therefore, a longitudinal enterotomy was performed to remove the foreign body which was a bolus of meat bezoar measuring (26.9 mm, 31.8 mm, 34.9 mm) (Fig. 4). The patient’s condition stabilized after the successful surgical procedure. Four days subsequent to the surgery, he was discharged home. After 18 months of follow-up, our patient experienced neither recurrence of small bowel obstruction nor complications related to the surgery.

## Discussion

Small bowel obstruction is a serious condition that needs careful management due to its difficult diagnosis and acute complications^1^. Many factors are reported in medical literature to cause SBO. Adhesions are considered the most frequent factor accounting for 73.8% of all SBO cases, while hernias are the second most frequent cause responsible for ~18.5% of all cases^6^. Other problems such as volvulus, intussusception, and tumours are rarely reported as SBO causing factors^1,4^.

Bezoar-induced small bowel obstruction is a rare condition that takes place in only 0.4–4.8% of SBO patients, and is usually found in the terminal ileum 50–70 cm proximal to the ileocecal valve due to its being the narrowest place in GIT^2–5^. Bezoars are classified into four main types based on their components: (1) Phytobezoars are masses of poorly digested vegetables or fruits with high amounts of cellulose or tannins, and are considered the most common type. (2) Trichobezoars are balls of hair, usually found within the stomach in psychiatric patients. (3) Pharmacobezoars are rarely reported cases of non-digested conglomerated medications. (4) Lactobezoars referring to an undigested mucus and milk mass in milk-fed infants^2,3,5,6^. Several types of bezoars such as metals, plastic, paper, fungi (*Candida*), and parasites are seldomly reported^3^. However, meat bolus bezoars are not classified under any established types of bezoars in the literature. Moreover, meat bolus bezoars are not named as phytobezoar because the term (Phyto) is derived from the Greek root (*φυτό*) that means plant^3^. Overall, phytobezoars’ components are only food materials that contain high levels of cellulose and/or tannin such as persimmons, mushroom, orange, and pumpkin as reported in many cases^2,3,5,6,8,9^. We would like to call this type of bezoar (Kreasobezoar), taking this term from the Greek root kreas (κρέας) which means meat.

In the literature, BI-SBO case reports are most frequently caused by phytobezoars^3^. However, meat bolus bezoar-induced-SBO is reported only by Henry *et al.* ^10^ after 12 years of Roux-en-Y gastric bypass surgery, and in Malhotra S^11^. where the meat bolus bezoar impacted the patient’s oesophagus. Generally, patients with previous gastric surgeries have an incidence of 5–12% to develop a bezoar within 9 months to 30 years later^1,3,5,10^. In fact, prior gastric surgeries are the most frequent risk factor of bezoar development in ~20–93% of BI-SBO patients, while other risk factors include postoperative adhesions, hypothyroidism, diabetes mellitus, Crohn’s disease, mental retardation, Guillain–*Barré* syndrome, myotonic dystrophy, pyloric dysfunction, gender (as Oh et al. found in their study that BI-SBO ratio is 3:1 male: female), and swallowing large volume food boluses due to inadequate mastication or bad dental health especially in old patients^1–3,5,10,12^. Our patient had not gone under gastric surgery but recalled a right inguinal hernia repair ~40 years ago, had a history of diabetes mellitus type II, and he wears a dental prosthesis which increases the possibility of bezoar formation in his GIT.

Symptoms of BI-SBO are manifold according to size of bezoar, location of impaction fundamentally. They include abdominal pain and distention as the main complaint, vomiting, nausea, dysphagia, constipation, satiety, and in some cases weight loss^4,5,12^. Our patient experienced gradual abdominal pain and distention, vomiting, and constipation mainly which matches the typical BI-SBO symptoms as Henry *et al.* ^10^ also reported their patient’s chief complaint was acute abdominal pain and emesis.

Clinical examination alone is insufficient for accurately distinguishing the underlying cause of small bowel obstruction; therefore, radiological imaging and studies play a crucial role in the diagnosis and management of SBO^1^. In our case, we initially obtained an AXR for the patient that revealed dilated intestinal loops with air-fluid levels, which raised suspicion of intestinal obstruction. However, a definitive diagnosis of bezoar existence cannot be established based on AXR findings in BI-SBO cases^1,3^. Consequently, to determine the underlying cause of the obstruction, we performed a contrast-enhanced CT scan of the patient’s abdomen and pelvis which also revealed dilated intestinal loops with a transitional area in the distal ileum and detected an intraluminal mass measuring (26.9 mm, 31.8 mm, 34.9 mm) which raised the suspicion of BI-SBO.

Radiologically, CT scan is an effective method for identifying the cause of acute SBO with high sensitivity 73–95% and specificity (up to 60%) by assessing small bowel loops’ features such as oedema, strangulation, ischaemia, or intra-abdominal fluid accumulation^1,3^. This procedure aids in determining the cause, location, and degree of the obstruction^1^. Therefore, the use of CT scan holds significance in preoperative planning as it usually precedes the surgical removal of intestinal bezoars within 48 h^2,3^. In this case, surgery was performed one day after the CT imaging. Henry *et al.* ^10^ performed a CT scan after the physical examination, and reported the same radiological features as ours. Oh *et al.* ^12^ mentioned that in medical literature ultrasonography (US) diagnostic rate in BI-SBO is 88–99%. However, in BI-SBO patients, US has a disadvantage due to impacted gas in GIT and being an operator-dependent procedure^12^. Thus, we claim that CT scan is the golden diagnostic standard in BI-SBO.

Treatment of BI-SBO is similar to other causes and etiologies of SBO: initially conservative therapy including fluid-electrolyte replacement and intestinal decompression. Eventually, there are four major approaches to bezoar management: (1) Chemical dissolution is usually used in upper GI bezoars. Chemicals used include saline solution, sodium bicarbonate, and even CocaCola. (2) Endoscopic fragmentation and removal that uses biopsy forceps, polypectomy snares, or electrosurgical knives. (3) Laparoscopy is frequently used nowadays despite its difficult performance due to intestinal dilation near obstruction location. Therefore, laparoscopy is only available in high-tech medical centers and should be performed by well-trained surgeons. (4) Laparotomy (open surgery) is the most used method to treat BI-SBO. Therapeutic approach is chosen depending on bezoar size, material, location, and accompanied pathology and complications^3–5^. With our patient, we initially chose conservative therapy. After 2 days of recording no improvement as well as studying the radiology imaging, we decided to directly proceed with open surgery. Henry *et al.* ^10^ chose diagnostic laparoscopy for assessing the bowels which appeared diffusely dilated. As they found laparoscopic procedure difficult to perform, they converted to open surgery and enterotomy.

## Conclusion

All in all, due to its hard diagnosis and serious complications, BI-SBO should be considered as a differential diagnosis when an emergency acute abdomen patient with SBO symptoms is present despite its rare occurrence and the type of bezoar. CT imaging is essential in the diagnosis of such cases as it is the most effective radiologic choice. First step of management includes conservative therapy. Surgical decision is mandatory if the conservative approach fails to enhance the patient’s condition. In the future, any type of ingested foreign bodies may be reported as a bezoar. Therefore, we recommend conducting updated studies and reviews. We would also suggest conducting additional research and reviews to explore the impact of age, diet, and dental health with the use of prosthesis in bezoar formation and BI-SBO.

### Limitation

The patient lost the abdominal X-Ray imaging file. Unfortunately, it was not found even after the extensive searching and looking for it. Another limitation is that since this is the second case report in the literature with this type of bezoar, there is a scarcity of information and resources for comparison.

## Ethical approval

Not applicable.

## Consent

Written informed consent was obtained from the patient for publication of this case report and accompanying images. A copy of the written consent is available for review by the Editor-in-Chief of this journal on request.

## Sources of funding

There are no funding sources.

## Author contribution

A.A. held the work concept and drafted the manuscript. F.K. participated in academic writing. W.A. extracted the references and assisted with academic writing. R.A.F. collected patient’s data and assisted with academic writing. A.F. performed the surgery and documented the patient’s follow-up. All authors revised the final manuscript and approved it.

## Conflicts of interest disclosure

The authors declare that they have no competing interests.

## Research registration unique identifying number (UIN)

Our research study does not involve human subjects.

## Guarantor

Ammar Albostani.

## Data availability statement

Since all data are included in the manuscript, there is no need data sharing.

## Provenance and peer review

Not commissioned, externally peer-reviewed.

## Consent for publication

All authors provide consent for publication.
